# The Importance of Large-Scale Genomic Studies to Unravel Genetic Risk Factors for Autism

**DOI:** 10.3390/ijms25115816

**Published:** 2024-05-27

**Authors:** Isabella de Sousa Nóbrega, André Luíz Teles e Silva, Bruno Yukio Yokota-Moreno, Andréa Laurato Sertié

**Affiliations:** Faculdade Israelita de Ciências da Saúde Albert Einstein, Hospital Israelita Albert Einstein, Rua Comendador Elias Jafet, 755. Morumbi, São Paulo 05653-000, Brazil; bol114@einstein.br (I.d.S.N.); andre.teles@einstein.br (A.L.T.e.S.); bol115@einstein.br (B.Y.Y.-M.)

**Keywords:** autism spectrum disorder, genomic technologies, large patient cohorts, locus discovery, gene discovery, genetic heterogeneity

## Abstract

Autism spectrum disorder (ASD) is a common and highly heritable neurodevelopmental disorder. During the last 15 years, advances in genomic technologies and the availability of increasingly large patient cohorts have greatly expanded our knowledge of the genetic architecture of ASD and its neurobiological mechanisms. Over two hundred risk regions and genes carrying rare de novo and transmitted high-impact variants have been identified. Additionally, common variants with small individual effect size are also important, and a number of loci are now being uncovered. At the same time, these new insights have highlighted ongoing challenges. In this perspective article, we summarize developments in ASD genetic research and address the enormous impact of large-scale genomic initiatives on ASD gene discovery.

## 1. Introduction

Autism spectrum disorder (ASD) affects approximately 1–2% of the world population [1,2] and is among the most heritable of developmental disorders, with an estimated heritability of about 50–90% [3,4,5,6]. Over the past 15 years, striking advances have been made in our understanding of the ASD allele architecture. This remarkable progress has been the result of (i) technological advancements in both high-density microarrays capable of identifying submicroscopic variation in chromosome structure (copy number variations, CNVs) across the genome, and high-throughput sequencing platforms enabling detection of genomic variants at base-pair resolution; (ii) the tremendous expansion of human genetic variation databases that allow for the identification of ultra-rare variants and the characterization of evolutionarily constrained genes intolerant to variation; (iii) the development of multiple scoring systems to estimate variant deleteriousness; (iv) the development of rigorous statistical methods to correct for multiple comparisons and overcome power limitations; and (v) the establishment of research initiatives to make biomaterials and genetic data from large patient cohorts with different designs (Box 1; Table 1) widely available to the scientific community.

Box 1 **Family-based and unrelated case control cohorts for genetic research in ASD.**

  Family-based studies can be mainly divided in simplex families, including one affected child and unaffected parents and siblings, and multiplex families, including two or more affected children and unaffected parents and siblings. ASD multiplex families comprise ~10% of affected families and are thought to have a different genetic architecture than simplex families. These family-based designs are particularly well suited for analysis of rare de novo and inherited high-impact variants. Also, the transmission information from parents to offspring allows investigation of parent-of-origin effects and transmission bias, which are not possible in case-control designs. The findings from large-scale family-based genomic studies provide most of what we know about specific genetic contributions to ASD.

  Unrelated case-control studies have been used to compare a group of unrelated ASD cases to a nonaffected population-based control group to identify both rare and common genetic risk factors that contribute to ASD. In the case-control design, large study populations can be assembled without the need to enroll also family members of the recruited participants. The results from a recent large-scale case-control genomic study provide information on common risk variants showing robust association with ASD.

**Figure 1:**
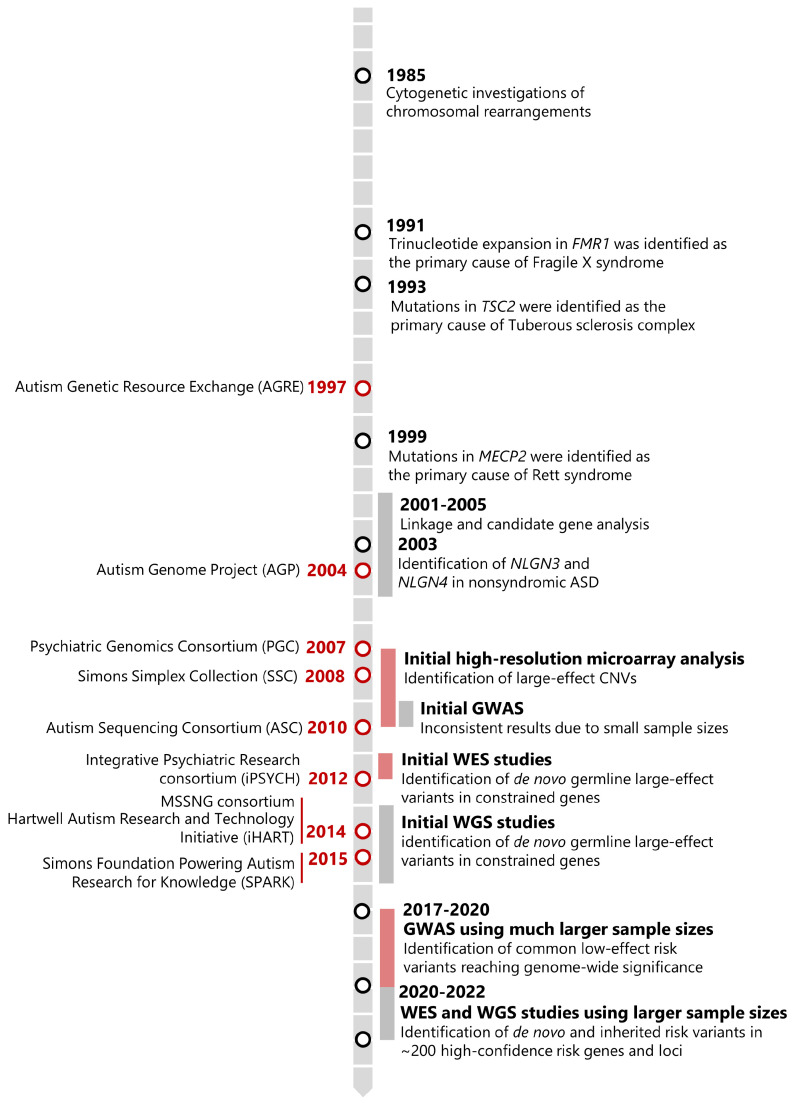
Timeline of key events in ASD genetic research and discovery. Over the past 40 years, genetic studies have provided crucial information on the genetic architecture of ASD. Methods and findings are depicted on the right, while large-scale cohort initiatives are indicated on the left. GWAS = genome-wide association studies; WES = whole exome sequencing; WGS = whole genome sequencing.

**Figure 2:**
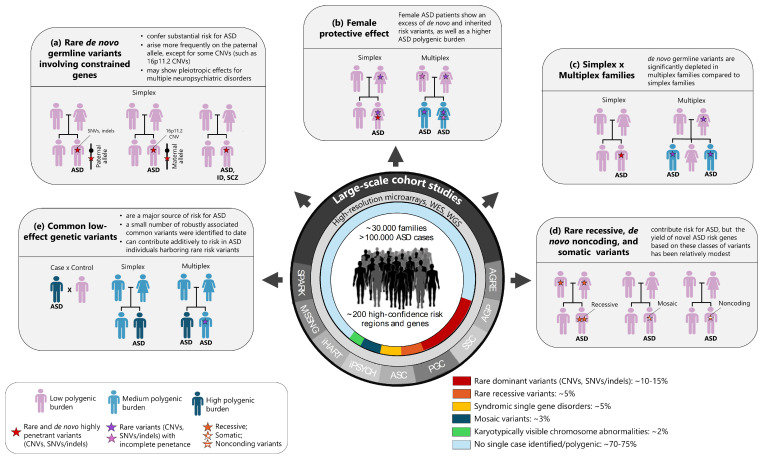
Main findings on the genetic architecture of ASD. Technical breakthroughs, including high-throughput microarrays and sequencing platforms, have enabled the search for a wide spectrum of risk variants via genome-wide surveys in increasingly large patient cohorts (see Table 1 for details). The main findings include the following: (**a**) Rare de novo germline CNVs, protein-truncating variants (loss-of-function SNVs and indels), and damaging missense variants involving constrained genes are overtransmitted to probands compared to siblings without ASD, arise more frequently on the paternal allele (except for some CNVs, such as 16p11.2 CNVs), and may show pleiotropic effects, thus increasing the risk for other complex neuropsychiatry disorders. (**b**) Female ASD patients from both simplex and multiplex families show an excess of de novo and inherited risk variants as well as a higher ASD polygenic burden, consistent with a female protective effect. (**c**) De novo germline CNVs and damaging SNVs/indels are significantly depleted in multiplex families compared to simplex families. (**d**) Rare recessive, de novo noncoding, and somatic variants also contribute to the risk for ASD; however, gene discovery based on these types of variants alone has been underpowered. (**e**) Common genetic variants of small individual effect sizes acting *en masse* carry the majority of the population risk for ASD; however, only a small number of common variants robustly associated with ASD have been identified to date. Furthermore, there is evidence that common and rare variations act additively to confer liability to ASD. WES = whole exome sequencing; WGS = whole genome sequencing; CNVs = copy number variants; SNVs = single-nucleotide variants; indels = insertions/deletions.

Results, mainly from family-based cohort studies, have led to the identification of more than 200 high-confidence risk regions and genes disrupted by rare, often de novo germline mutations of large effect [18,19,20,21]. Additionally, recent results from genome-wide association studies (GWAS) involving very large family-based and case-control samples have successfully identified common risk alleles of small effect, reaching genome-wide significance [17,22,23]. This is consistent with high genetic heterogeneity, including the contribution of large-effect rare variants and many low-effect common variants through polygenic risk [24,25,26]. In this review, we focus on how the advances in genomic technologies, along with the collection of increasingly large patient cohorts, have offered complementary data and contributed to our understanding of the genetic causes of ASD. Figure 1 shows a timeline of key events in the history of genetic research in ASD, and Figure 2 highlights the main findings on ASD genetics to date.

## 2. Delineation of Locus and Gene Discovery in ASD

### 2.1. Chromosomal Rearrangements and Monogenic Syndromes Associated with ASD

The first insights into the genetic causes of ASD came from cytogenetic studies of chromosomal abnormalities [27], and from the identification of rare monogenic syndromes with high penetrance for ASD, often referred to as “syndromic ASD”, including Fragile X syndrome [28,29,30], Tuberous sclerosis complex [31,32], and Rett syndrome [33]. While conventional cytogenetic analysis failed to accurately associate a specific gene with ASD because of the low resolution of the karyotypes (~5 Mb), the study of the ASD-related monogenic syndromes provided important initial clues regarding altered biological functions in ASD, including RNA binding, the mammalian target of the rapamycin (mTOR) pathway, and chromatin modification. These early findings were supported by later large-scale genomic studies examining idiopathic ASD cohorts.

### 2.2. Linkage and Candidate Gene Analysis

The initial efforts to identify genes associated with idiopathic ASD have undertaken family-based studies, using mainly multiplex family samples (n = ~110–345 families) such as those from the Autism Genetic Resource Exchange (AGRE) collection (Table 1), focused on the following: (i) linkage analysis to identify genomic regions commonly inherited by affected family members [34,35,36]; and (ii) candidate gene association studies to find common variants associated with ASD within a limited number of pre-specified genes [37,38,39]. Although initially thought to be sufficiently powered, these investigations have been hindered by the small sample sizes and sparse marker sets used, resulting in a lack of reproducible results.

The first step towards the successful identification of genes associated with ASD that do not occur in the context of a clinically defined monogenic syndrome was taken in 2003. Through traditional cytogenetic mapping strategies and parametric linkage analysis, followed by targeted DNA sequencing, Jamain et al. [40] identified disruptive variants in two genes involved in synaptic function, *NLGN3* and *NLGN4*, in individuals with nonsyndromic ASD.

### 2.3. Microarray-Based Copy Number Variation Analysis

The discovery of rare and recurrent CNVs as important contributing factors in ASD susceptibility was a watershed moment in ASD genomics. From the late 2000s onwards, several studies have applied high-density microarrays in increasingly large samples of simplex and multiplex families (from ~260 to ~2450 families), mainly from the AGRE, Autism Genome Project (AGP), and Simons Simplex Collection (SSC) initiatives (Table 1), leading to an era of reproducible findings in the genetic architecture of ASD. Collectively, these studies found a significantly higher frequency of large CNVs that disrupted gene-containing regions of the genome, both de novo and inherited, in ASD cases compared to controls; they were present in 5–10% of affected individuals, compared to <1–2% in unaffected controls [41,42,43,44,45,46,47,48]. Recurrent large CNVs include loci at 1q21.1, 2p16.3, 3q29, 7q11.23, 15q11-13, 15q13.3, 16p11.2, 22q11.2, and 22q13.3. Many CNVs overlap with ASD-risk genes encoding synaptic proteins (such as *NRXN1*, *SHANK3*, *SYNGAP1*, and *NLGN3*), providing supporting evidence that synaptic dysfunction may be involved in the neuropathology of ASD. Certain loci are recurrently found to be duplicated or deleted in ASD, including 16p11.2, 15q11-13, and 22q11.2. It was also observed that simplex families have a slightly greater burden of de novo CNVs than multiplex families [41,47,48], and that de novo CNVs are correlated with lower IQ and are overrepresented in females in both simplex and multiplex families [47,48], supporting the notion of sex-specific thresholds where females require a higher genetic load to express ASD (called the female protective effect) [49]. Furthermore, non-sharing pathogenic CNVs were found in members of multiplex families, providing further evidence of genetic heterogeneity in ASD even within multiplex families [48]. Finally, it was also observed that most ASD-associated CNVs show pleiotropic effects for multiple neuropsychiatric disorders, including epilepsy, schizophrenia, and intellectual disability [45,47].

### 2.4. Whole Exome and Genome Sequencing

The evolution of high-throughput genomic sequencing has transformed ASD gene discovery. In 2011–2012, five studies applied whole exome sequencing (WES) to simplex families, mainly from the SSC cohort (a combined total of 965 families), and found a statistically significant excess of de novo, putative loss-of-function variants (including nonsense, frameshift insertions/deletions, and canonical splice sites—also referred to as protein-truncating variants) in brain-expressed genes in ASD probands [50,51,52,53,54]. Moreover, it was observed that de novo damaging missense variants also carry risk, but with an overall effect size that is smaller than for putative loss-of-function variants [50,51,52]. Another important finding was that de novo variants are predominantly of paternal origin and positively correlated with paternal age [52]. Some genes, such as *SCN2A*, *GRIN2B*, and *CHD8*, were found to show recurrent protein-altering variants across these studies.

Successive WES studies using larger patient cohorts and novel statistical methods to overcome power limitations (such as the “transmitted and de novo association” [TADA] method, which incorporates information from multiple variant classes and produces gene-level measures of evidence for association that can be transformed into a false discovery rate [FDR]—[55]) have been successfully performed to confirm previous results and aid gene and locus discovery in ASD [18,56,57,58,59,60]. The largest WES study to date included 49,050 family-based samples (15,036 ASD cases) and 14,118 case-control samples (5591 ASD cases) from the Autism Sequencing Consortium (ASC), SSC, and the recently released Simons Foundation Powering Autism Research for Knowledge (SPARK) initiative (Table 1), and identified 72 ASD risk genes at FDR ≤ 0.001, and 185 genes at FDR ≤ 0.05 [19]. Importantly, it has been shown that the high-confidence ASD risk genes are more likely to be intolerant to loss-of-function variants (more constrained), are expressed at relatively higher levels in maturing excitatory neurons at specific embryonic prefrontal cortex regions and layers, and have roles in the regulation of gene expression (e.g., *CHD8*, *ADNP*, *FOXP1*, *CHD2*, *POGZ*, *KMT5B*) and neuronal communication (e.g., *SCNA2*, *SHANK3*, *SYNGAP1*, *PTEN*, *GRINB2*, *NRXN1*), thereby highlighting the functional convergence of genes associated with ASD [18,19,61,62,63,64]. Additionally, similar to ASD-related recurrent CNVs, several ASD-related genes have pleiotropic effects, being involved in multiple stages of brain development and contributing to a broad range of neurodevelopmental disorders in addition to ASD [18,19]. Finally, it is also noteworthy that analysis of WES data from the ASC and SSC initiatives has uncovered reliable risk for ASD from rare recessive [65] and somatic variants [66,67,68,69]. However, the yield of novel ASD risk genes based on these classes of variants has been relatively modest.

**Figure 3:**
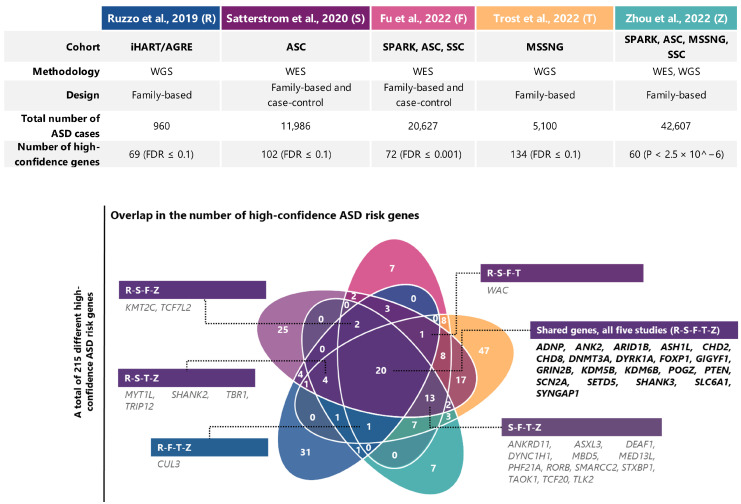
Summary of recent exome and genome sequencing studies using different cohort designs for gene discovery in ASD. Overview of five recent large-scale WES and WGS studies: Ruzzo et al., 2019 (**R**) [77]; Satterstrom et al., 2020 (**S**) [18]; Fu et al., 2022 (**F**) [19]; Zhou et al., 2022 (**Z**) [21]; Trost et al., 2022 (**T**) [20]. The cohorts, experimental designs, total number of ASD cases analyzed, and the total number of high-confidence ASD risk genes identified [false discovery rate (FDR) ≤ 0.001–0.1 or *p* < 2.5 × 10^−6^] are shown. Together, these studies identified a total of 215 different high-confidence ASD risk genes. The Venn diagram shows the overlap in the number of high-confidence risk genes detected by these studies, highlighting the extreme genetic heterogeneity of ASD. Shared risk genes identified by at least four studies are shown.

Whole genome sequencing (WGS) technology offers the ability to sequence the entire genome with more uniform coverage than microarrays and WES, allowing the detection of all classes of genetic variation in the coding and noncoding regions. Initial WGS studies, conducted during 2013–2016 and involving 32–200 families, revealed similar findings of excessive de novo protein-truncating variants in ASD, but showed some inconsistent findings about risk loci in the noncoding genome [70,71,72,73]. Subsequent WGS studies in larger cohorts including both simplex and multiplex families—such as those from the SSC, MSSNG, and AGRE/iHart initiatives (Table 1)—have succeeded in capturing a broad array of de novo and inherited ASD risk variants that affect the coding sequence and cis-regulatory elements [15,74,75,76,77,78,79,80,81]. More recently, the MSSNG initiative analyzed WGS data from 4258 families (5100 individuals with ASD and 6212 unaffected parents and siblings) and, after incorporating WES data from the SPARK cohort to increase power, identified 134 ASD-associated genes with FDR ≤ 0.1, including 67 previously unrecognized risk genes, as well as numerous rare structural and mitochondrial ASD-associated variants [20]. Furthermore, in contrast to previous results [75], limited enrichment of rare variants impacting noncoding elements was observed in individuals with ASD, which highlights the challenges in accurately detecting noncoding risk variants [20]. Finally, Zhou et al. (2022) [21] aggregated WES and WGS data of over 29,000 families (42,607 ASD cases) from the SPARK, ASC, MSSNG, and SSC initiatives and, aside from confirming earlier findings, reported only five new risk genes supported by both de novo and inherited rare coding variants, and suggested that new ASD genes to be discovered are likely to have smaller effect sizes and require even larger sample sizes than known, highly penetrant ASD genes. Figure 3 provides a summary of recent large-scale WES and WGS studies using cohorts with different designs for gene discovery in ASD, with a description of the high-confidence ASD risk genes shared among these studies.

### 2.5. Genome-Wide Association Studies (GWAS)

GWAS compares common single-nucleotide polymorphisms (SNPs with minor allele frequency >1% in the population) between cases and controls to test for association with a specific trait or disease. Even though there is strong evidence that common variants of small effect, acting additively, account for a major part of ASD liability [3,82], initial GWAS in ASD using more than 1500 cases, mainly from families of AGRE and AGP collections, produced inconsistent results due to insufficient statistical power [83,84,85,86,87]. However, more recent studies involving much larger sample sizes—such as those from the Integrative Psychiatric Research (iPSYCH) Danish case-control cohort, the Psychiatric Genomics Consortium (PGC), and the SPARK initiative (Table 1)—have successfully identified common alleles robustly associated with ASD [17,22,23]. A landmark study by Grove et al. (2019) [17] combined GWAS data from 18,381 individuals with ASD and 27,969 controls from the iPSYCH and PGC collections, and found five common genetic variants reaching genome-wide significance, with a further seven loci shared with other psychiatry disorders (schizophrenia, major depression, and educational attainment) also reaching the genome-wide threshold, thereby implicating a shared genetic background in neuropsychiatry conditions. Subsequent studies have confirmed and extended these findings [88,89].

One increasingly popular application of GWAS results relies on polygenic risk scores (PRSs), which are estimations of a person’s disease risk based on the combined impact of common variants. Despite the relatively small number of common alleles associated with ASD to date, PRSs have already demonstrated a modest ability to differentiate between cases and controls [17,20,24,26,90]. Furthermore, PRSs appear to differ by sex, with a higher genetic load detected in females, consistent with a female protective effect [26], and, surprisingly, have been associated with cognitive aptitude [17,90,91]. Moreover, it was found that polygenic variation also contributes additively to risk in ASD individuals harboring large-effect variants, consistent with the model that the polygenic background can modulate the effect of rare mutations with large effects [24,25,92].

## 3. Challenges and Future Directions

Advancements in genomic technologies and the creation of large patient cohorts and genomic datasets via public and privately funded resources have led to a better understanding of the genetic contributions to ASD. This includes identifying common genetic variants robustly associated with ASD and over 200 high-confidence risk regions and genes. Despite this, much remains unknown. Given the small effect size of individual common variants, dissecting the effects of these variants and understanding how they interact with each other, and with environmental risk factors, to converge on biological networks contributing to ASD is not a simple task. Additionally, the interplay between common and rare variants remains poorly understood, and the predictive ability of PRSs is too low to be clinically useful. Many risk CNVs, which are now one of the most robustly replicated features of the genetic architecture of ASD, can involve intervals containing multiple genes, and dissecting the contribution of any single gene within these intervals is challenging. Functional interpretation of noncoding variants also represents a persistent challenge. Rare variants carrying a large risk for ASD in specific genes have been particularly important in advancing our understanding of the underlying mechanisms. However, these variants account for a small percentage of the overall cases, and predictive modeling suggests that there are ~1000 genes with disruptive variants associated with ASD [55,93], indicating that further work is required to fully elucidate genes and biological networks contributing to ASD. Moreover, many of the large-effect variants implicated in ASD show incomplete penetrance and pleiotropic biological effects, which hamper the identification of the relevant pathobiology among many potential downstream perturbations. Finally, despite the success in identifying multiple types of genetic risk, an effective treatment for the core symptoms of ASD has not yet been discovered and used in clinical practice. Therefore, identifying the etiological mechanisms in the individuals with ASD who remain genetically unresolved, and translating lists of risk regions and genes into reliable understanding of the underlying neurobiology to ultimately improve diagnoses and clinical care for patients and families remain major challenges for the field.

**Figure 4:**
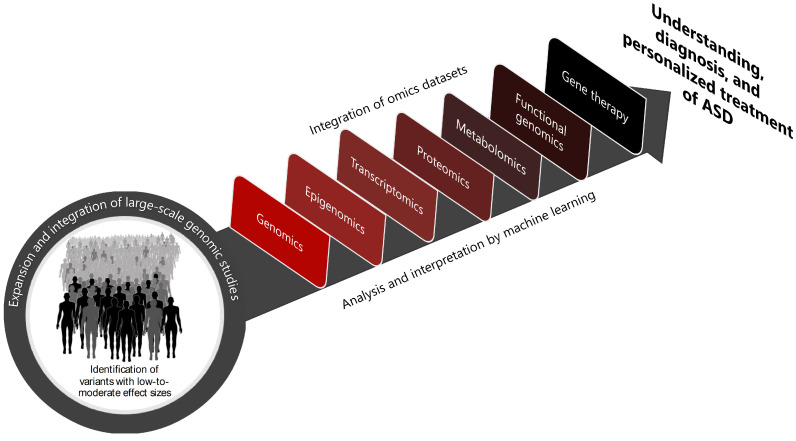
Future directions for research and clinical care in ASD. Data from much larger sample sizes of patients and their families will facilitate the identification of the full spectrum of genetic variations associated with ASD. The integration of genomics with other omics datasets (such as epigenomics, transcriptomics, proteomics, metabolomics, and functional genomics) and the application of machine learning algorithms to combine multi-omics data and predict risk genes and treatment outcomes will offer unprecedented possibilities to enhance the current understanding of the causes and mechanisms of ASD and advance diagnosis and personalized treatment.

Yields from ASD GWAS and rare variant analysis, mainly those rare variants with moderate effect sizes, are expected to continue to increase as sample sizes increase and as genomic technologies continue to evolve. The integration of omics datasets (such as genomics, epigenomics, transcriptomics, proteomics, metabolomics, and other emerging omics) generated from the autistic and typically developing human brains will allow a more exhaustive comprehension of these processes at the molecular, cellular, and systemic levels, expanding the ability to contextualize the role of ASD risk genes. Functional genomic approaches to interrogate the functional consequences of perturbing ASD risk genes, such as those involving CRISPR-based genetic screens both in model organisms [94,95] and in human pluripotent stem cell-derived neurons [64,96] and brain organoids [97,98], will also accelerate research progress in ASD. CRISPR-mediated gene therapy, such as CRISPR activation (CRISPRa) to recover the haploinsufficiency of ASD risk genes, has recently been used in preclinical studies [99,100] and holds great promise for the future of ASD treatment. Sophisticated computational approaches such as machine learning have the potential to predict risk genes using human brain-specific spatiotemporal gene expression signatures and protein–protein interaction networks, thus anticipating the trajectory of gene discovery in ASD [101,102,103,104]. Ultimately, these strategies have the potential to broaden the understanding of ASD pathobiology and direct future therapeutic efforts for groups of patients with distinct etiologies (Figure 4).

## Figures and Tables

**Table 1:** Key large-scale cohorts/consortium initiatives that have contributed to the identification of genetic risk factors for ASD.

Cohort/Consortium Studies	References and Website	History	Study Design	Current Size	Genomic Datasets
Autism Genetic Resource Exchange (AGRE)	[7,8] https://www.autismspeaks.org/about-agre (accessed on 20 May 2024)	Established in 1997 by Cure Autism Now (CAN), AGRE is currently funded by the National Institute of Mental Health and Autism Speaks, which merged with CAN in 2006. AGRE houses a collection of biomaterials and genetic and phenotypic data from families with ASD, made available to the greater scientific community	Simplex and multiplex families	~2000 families	Genome-wide genotyping, WES, WGS
Autism Genome Project (AGP) ^a^	[9] https://www.ncbi.nlm.nih.gov/projects/gap/cgi-bin/study.cgi?study_id=phs000267.v5.p2 (accessed on 20 May 2024)	Established in 2004 by the National Alliance for Autism Research and the National Institutes of Health, AGP is now coordinated by Autism Speaks. The project involves more than 50 centers in North America and Europe that have pooled their DNA samples in a collaborative effort to examine families for SNPs and CNVs affecting risk for ASD	Simplex and multiplex families	~2600 families	Genome-wide genotyping
Simons Simplex Collection (SSC)	[10] https://www.sfari.org/resource/simons-simplex-collection/ (accessed on 20 May 2024)	Established in 2008 by the Simons Foundation Autism Research Initiative, SSC is a repository of clinical and genetic data from simplex ASD families designed to facilitate the discovery of rare and de novo variation	Simplex families	~2600 families	Genome-wide genotyping, WES, WGS
Psychiatric Genomics Consortium (PGC)–ASD Working Group	[11,12] http://www.med.unc.edu/pgc (accessed on 20 May 2024)	Founded in 2007 by Dr. Patrick Sullivan with the aim of conducting statistically rigorous and comprehensive GWAS meta-analysis for common psychiatric disorders. The ASD Working Group was founded at the start of PGC, in 2008, and currently includes 48 investigators from 10 countries	Simplex families, case-control	~18,000 ASD cases ^b^	Genome-wide genotyping
Autism Sequencing Consortium (ASC) ^c^	[13] https://genome.emory.edu/ASC/ (accessed on 20 May 2024)	Founded in 2010 by Dr. Joseph D. Buxbaum, the ASC is a large-scale international genomic consortium integrating several ASD cohorts and WES data from over 100 investigators. The project is funded by the National Institute of Mental Health with additional support from the National Human Genome Research Institute	Simplex families, case-control	~12,000 ASD cases	WES
Integrative Psychiatric Research (iPSYCH)	[14] https://ipsych.dk/en/about-ipsych (accessed on 20 May 2024)	Founded in 2012 by six leading Danish researchers in the fields of psychiatry and genetics, the project consists of a large Danish population-based case-cohort sample aimed at unraveling the genetic and environmental architecture of five of the most serious mental disorders (schizophrenia, ASD, ADHD, bipolar disorder, and depression)	Case-control	~15,000 ASD cases ^d^	Genome-wide genotyping, WES
Hartwell Autism Research and Technology Initiative (iHART)/AGRE ^e^	https://thehartwellfoundation.com/iHART.shtml (accessed on 20 May 2024)	Launched in 2014 by the Hartwell Foundation, the iHART database contains WGS data from multiplex families of the AGRE collection	Multiplex families	~1000 families (~4500 ASD cases	WGS
MSSNG ^a^	[15] https://research.mss.ng/ (accessed on 20 May 2024)	Launched in 2014, the project is a collaboration between Autism Speaks, Verily, DNAstack, Hospital for Sick Children, and the research community. Its primary goal was to create the world’s largest WGS database of 10,000 individuals with ASD and their families with deep phenotyping	Simplex and multiplex families	4258 families (5100 ASD cases)	WGS
Simons Foundation Powering Autism Research for Knowledge (SPARK)	[16] https://www.sfari.org/resource/spark/ (accessed on 20 May 2024)	Established in 2015 by the Simons Foundation Autism Research Initiative, the project aims to establish a permanent repository of genetic samples from 50,000 families with ASD living in the U.S.	Simplex and multiplex families	~50,000 ASD cases	Genome-wide genotyping, WES, WGS

^a^. The AGP and the MSSNG consortium include samples from AGRE, as well as from other initiatives that were not presented here. ^b^. Current sample size is not available at the cohort website. Numbers were based on [12,17]. ^c^. The ASC initiative includes samples from SSC, AGRE, and iPSYCH, as well as from other initiatives that were not presented here. ^d^. Current sample size is not available on the cohort website. Numbers were based on [14]. ^e^. The iHART initiative includes samples from AGRE.
